# Seeing the forests *and* the trees—innovative approaches to exploring heterogeneity in systematic reviews of complex interventions to enhance health system decision-making: a protocol

**DOI:** 10.1186/2046-4053-3-88

**Published:** 2014-08-12

**Authors:** Noah Ivers, Andrea C Tricco, Thomas A Trikalinos, Issa J Dahabreh, Kristin J Danko, David Moher, Sharon E Straus, John N Lavis, Catherine H Yu, Kaveh Shojania, Braden Manns, Marcello Tonelli, Timothy Ramsay, Alun Edwards, Peter Sargious, Alison Paprica, Michael Hillmer, Jeremy M Grimshaw

**Affiliations:** 1 Department of Family and Community Medicine, Women’s College Hospital-University of Toronto, 77 Grenville Street 4th Floor, Toronto, Ontario M5S 1B3, Canada; 2 Li Ka Shing Knowledge Institute of St. Michael’s Hospital, 30 Bond St, Toronto, Ontario M5B 1W8, Canada; 3 Department of Health Services, Policy & Practice and Center for Evidence-based Medicine, School of Public Health, Brown University, Providence, RI 02912, USA; 4 Clinical Epidemiology Program, Ottawa Hospital Research Institute. Centre for Practice-Changing Research, The Ottawa Hospital-General Campus, 501 Smyth Road, Box 711, Ottawa, Ontario K1H 8L6, Canada; 5 Epidemiology & Community Medicine, Department of Medicine, University of Ottawa, 451 Smyth Road, Room 3105, Ottawa, Ontario K1H 8M5, Canada; 6 PPD/CHEPA, McMaster University, 1280 Main St. West, Hamilton, Ontario L8S 4K1, Canada; 7 Department of Medicine, Sunnybrook Health Sciences Centre, University of Toronto Centre for Quality Improvement and Patient Safety (C-QuIPS), 2075 Bayview Ave., Room H468, Toronto, Ontario M4N 3M5, Canada; 8 Department of Community Health Services, University of Calgary, 3330 Hospital Drive NW, Calgary, Alberta T2N 4N1, Canada; 9 Department of Medicine, University of Calgary, 1403-29th St NW, Calgary, Alberta T2N 2T9, Canada; 10 Division of Nephrology, Department of Medicine, University of Calgary, 7th Floor, TRW Building, 3280 Hospital Drive NW, Calgary, Alberta T2N 4Z6, Canada; 11 Institute of Health Policy, Management and Evaluation, University of Toronto, 155 College Street, Suite 425, Toronto, Ontario M5T 3M6, Canada; 12 Ontario Ministry of Health and Long-Term Care, 80 Grosvenor Avenue, M7A 1R3, Toronto, Ontario, Canada

**Keywords:** Diabetes care, Knowledge translation, Quality improvement interventions, Complex Interventions, Health system decision-makers, Systematic review, Meta-analysis, Implementation science, Heterogeneity, Hierarchical modeling

## Abstract

**Background:**

To improve quality of care and patient outcomes, health system decision-makers need to identify and implement effective interventions. An increasing number of systematic reviews document the effects of quality improvement programs to assist decision-makers in developing new initiatives. However, limitations in the reporting of primary studies and current meta-analysis methods (including approaches for exploring heterogeneity) reduce the utility of existing syntheses for health system decision-makers. This study will explore the role of innovative meta-analysis approaches and the added value of enriched and updated data for increasing the utility of systematic reviews of complex interventions.

**Methods/Design:**

We will use the dataset from our recent systematic review of 142 randomized trials of diabetes quality improvement programs to evaluate novel approaches for exploring heterogeneity. These will include exploratory methods, such as multivariate meta-regression analyses and all-subsets combinatorial meta-analysis. We will then update our systematic review to include new trials and enrich the dataset by surveying authors of all included trials. In doing so, we will explore the impact of variables not, reported in previous publications, such as details of study context, on the effectiveness of the intervention. We will use innovative analytical methods on the enriched and updated dataset to identify key success factors in the implementation of quality improvement interventions for diabetes. Decision-makers will be involved throughout to help identify and prioritize variables to be explored and to aid in the interpretation and dissemination of results.

**Discussion:**

This study will inform future systematic reviews of complex interventions and describe the value of enriching and updating data for exploring heterogeneity in meta-analysis. It will also result in an updated comprehensive systematic review of diabetes quality improvement interventions that will be useful to health system decision-makers in developing interventions to improve outcomes for people with diabetes.

**Systematic review registration:**

PROSPERO registration no. CRD42013005165

## Background

There is a consistent evidence of gaps between what research evidence suggests is optimal care and the actual care provided by health care professionals and health systems [1-3]. The increasing recognition of such gaps has led to greater policy interest in evidence-based quality improvement (QI) programs to improve quality of care and subsequent patient outcomes. In fact, Ontario’s Ministry of Health, a large Canadian health care system, has structures and processes in place to support the use of systematic reviews in health system decision-making [4]. However, systematic reviews of QI programs usually include highly heterogeneous studies addressing varied interventions that are implemented differently across diverse health care contexts, rendering results obtained with standard meta-analysis methods difficult to interpret [5].

Several methodological issues must be addressed to enhance the utility of systematic reviews of QI programs. First, QI programs usually involve multifaceted approaches that can contain a mix of effective and ineffective (or even harmful) component QI interventions, which can interact (synergistically or antagonistically) among themselves. Understanding the effectiveness of each component of a QI program is a prerequisite for informing program development [6]. Second, the mechanisms of action of QI programs (and component interventions) are poorly understood, resulting in a lack of consensus regarding taxonomies of QI programs and component interventions [7]. As a result, authors of syntheses often need to develop operational, and somewhat arbitrary, classifications of the programs and interventions of interest. Misclassifying programs or interventions can add ‘noise’ in meta-analysis by artificially increasing variation in treatment effect estimates [e.g., leading to heterogeneity in post-treatment glycosylated hemoglobin (HbA1c) mean differences]. Furthermore, the use of arbitrary classification systems makes developing sensitive literature searches for the systematic review challenging. This likely results in reduced precision, as studies that evaluate a relevant intervention are inadvertently missed. Third, the effects of QI programs are likely modified by poorly recognized contextual factors and participant characteristics, making judgments about the applicability of the effects of interventions in different contexts and populations more challenging [8,9]. Finally, poor reporting of information on intervention- and context-related factors in the primary studies (e.g., how health care is financed, organized, and delivered in settings where complex interventions are evaluated) exacerbates this issue [10-12].

Systematic review authors have come to expect substantial heterogeneity when conducting syntheses of QI programs. In such cases, estimating the ‘mean effect’ of interventions is often inadequate because it averages over potentially important data patterns [13]. Specifically, the meta-analytic average may hide that some component interventions are highly effective and some are not (or are even harmful). Understanding which components are likely to have the greatest effect in a given scenario is crucial, particularly when resources available for implementation are scarce. Furthermore, when meta-analysis is used to estimate the ‘average’ effect across studies, potential effect modification by contextual factors may be overlooked, despite the importance of this information for health system decision-makers. Contextual factors can affect the generalizability and scalability of QI interventions and thus limit decision-makers’ willingness or ability to implement them [14].

Current approaches to exploring heterogeneity in systematic reviews of QI programs are relatively limited and typically involve the examination of statistical, clinical, and methodological heterogeneity with simple meta-analytic methodologies (e.g., examining the consistency of effect sizes across included studies, subgroup analyses, and univariate meta-regression analyses) [15]. In addition, typical analyses do not allow decision-makers to predict the effectiveness of combinations of QI components that have not yet been evaluated in trials. We view the presence of heterogeneity as an opportunity [13] to more directly address the questions of interest to health system decision-makers.

### Example: syntheses on strategies for quality improvement in diabetes

Diabetes is a chronic disease with high impact in terms of health care resource utilization, costs, societal impact, and health outcomes [16-18]. We recently published a systematic review of 12 QI interventions for diabetes [19]. As of July 2010, we identified 142 randomized controlled trials (RCTs) evaluating QI programs, of which 125 involved multi-component programs. Using data from the included RCTs, we performed random effects meta-analyses of QI programs (including one or more component interventions) versus usual care. The summary effect size for each outcome of interest (e.g., the mean difference in HbA1c) expressed the efficacy of QI programs that included the component of interest compared to QI programs that did not include the component of interest (e.g., QI programs including case management compared to QI programs not including case management, regardless of the presence of other components). While easier to specify, this meta-analytical approach did not directly model the effect of each QI component or assess interactions among components. In addition, our previous study did not use all available information. For example, in multi-arm RCTs, only the comparison of one of the experimental arms versus usual care was used to avoid duplication of information [20]. Although we observed improvements across virtually all outcomes [19], we were unable to distinguish the effectiveness of different QI components and we did not fully assess potential effect modifiers. Meta-regression adjusting for median baseline HbA1c values (<8.0% vs. ≥8.0%) and median effective sample size (≤141 patients vs. >141 patients) also did not distinguish between the effectiveness of different QI strategies. However, subgroup analyses indicated that effect sizes for QI strategies varied according to patients’ baseline diabetes control, suggesting the need for further work to identify additional effect modifiers. Unfortunately, reporting of intervention-related details and contextual information was poor, and the quality of study reporting did not improve over time [21].

In summary, despite a significant body of research that continues to expand considerably each year, we were unable to effectively use published data and current synthesis methods to adequately explore heterogeneity to address the health system-relevant questions regarding various QI strategies for diabetes care.

### Innovative meta-analytical approaches to explore heterogeneity in systematic reviews

To address the issue of complexity in systematic reviews of multi-component interventions, Noyes et al. argue for the development and application of new synthesis methods, including methods that can assess interactions between components [9]. For inference on the comparative effectiveness of complex interventions, such as those examined in the review of diabetes QI strategies, hierarchical multivariate meta-regression methods allow the specification of models that better reflect the structure of the data [22-24]. For example, using multivariate meta-analysis methods, we can evaluate the effect of QI components (or other arm-level factors) on the outcome of interest (e.g., mean HbA1c) within each arm of each RCT (level I: within study) and then model between-study variability to account for unexplained heterogeneity (level II: between-studies). The resulting multivariate meta-regressions would allow for the exploration of interactions between component interventions and effect modifiers [25-27]. They would also facilitate the inclusion of information from multi-arm trials and trials that compare active interventions (i.e., head-to-head comparisons without a usual care comparator).

For further data exploration, all-subsets combinatorial meta-analysis can be used to visualize and explore heterogeneity [28]. This involves performing a meta-analysis on all possible subsets of the available studies in a meta-analysis. For example, in a meta-analysis of three studies (*a*, *b*, *c*) there are seven possible subsets (*a*, *b*, *c*, *ab*, *ac*, *bc*, *abc*). In an all-subsets meta-analysis, summary effect sizes are calculated for each of these subsets. If the studies are homogeneous, similar results are obtained, regardless of the subset chosen. On the other hand, if there is heterogeneity, some subsets will show different results than others. For example, if two influential studies, *a* and *b*, have very different (and adequately precise) estimates, then subsets that include a but not b (e.g., *a* and *ac*) would yield different results compared to subsets that include b but not a (e.g., *b* and *bc*). Results in the subset including both studies (*abc*) will also produce different results (and will exhibit substantial heterogeneity). When the all-subsets meta-analyses have been completed, various explanatory graphs can be generated to assist interpretation, including a histogram of the frequency distribution of summary effect sizes and scatterplots of effect sizes over *I*^2^ statistics. All-subsets meta-analysis provides a rich set of data through which both deductive and inductive questions can be explored. Specifically applied to our data, we could deductively explore the robustness of the commonly used QI taxonomy by exploring the degree to which studies evaluating QI programs with specific component interventions appear relatively homogeneous with each other or whether there are heterogeneous clusters that vary with respect to specific characteristics of interventions, co-interventions, or contexts (e.g., health care settings, targeted health professionals, patient characteristics). We could also inductively explore variables across studies to identify potential key effect modifiers (i.e., characteristics shared among homogeneous clusters of studies that have higher or lower than average effect sizes).

### Enriching data to enhance the utility of syntheses

Due to a variety of reasons (e.g., author preferences, journal editorial policies), detailed descriptions of complex interventions and the contexts in which they are employed are rarely provided in published papers [9]. In such cases, it may be necessary to review other reports pertaining to the same study or to contact the authors of the primary studies to obtain additional information. For example, in a synopsis of high-quality trials and systematic reviews published in *Evidence-Based Medicine* between 2005 and 2006, Glasziou and colleagues observed that only 29% of descriptions of non-drug treatments were detailed enough to replicate in practice [29]. Authors of this study were able to supplement published descriptions through related publications or contact with authors, improving reporting completeness to around 65%. Contacting authors is not routinely undertaken in systematic reviews, perhaps because of the substancial resources required to complete such a task.

### Objectives

The aims of this project are as follows:

• To update our systematic review and to determine the effectiveness of QI strategies on diabetes quality of care

• To explore the use of novel meta-analytical techniques to enhance the utility of systematic reviews of complex multi-component interventions for health system decision-makers

• To explore the feasibility and value of surveying primary study authors to enrich the utility of systematic reviews of complex multi-component interventions for health system decision-makers

• To engage in extensive integrated and end-of-grant knowledge translation (KT) activities targeting key stakeholders in Canada and beyond

## Methods/Design

**Figure 1:**
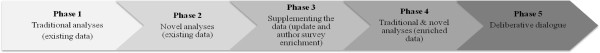
Phases of the forest and trees study.

To address the objectives above, this project will be conducted in five phases (1 through 5) (Figure 1). Briefly, phase 1 will explore the value of additional analyses using traditional meta-analytic techniques (i.e., meta-regression and subgroup analyses) on the existing dataset. Phase 2 will explore the value of novel meta-analytic techniques on the existing dataset. Phase 3 will supplement the existing dataset by updating the search, extracting additional variables pertinent to context and intervention, and performing a tailored author survey to enrich the data and improve completeness of all variables. Phase 4 will apply traditional and novel meta-analytic techniques on the enriched and updated data set. Phase 5 will develop and convene a ‘deliberative dialog’ with key stakeholders to consider the implications of the findings from the systematic review. To complete these tasks, we have adopted an integrated KT approach.

### Integrated KT approach

The degree to which we will successfully complete our project’s objectives is predicated on the extent to which we understand the needs of health care decision-makers. The integrated KT approach engages knowledge users in planning, conducting, and interpreting a synthesis [30-32], to facilitate understanding of their needs. For our purposes, we defined knowledge users as individuals who are ‘likely to be able to use the knowledge generated [from this project] in order to make informed decisions about health policies, programs and/or practices’ [33]. Integrated KT approaches have evolved out of traditions of collaborative research and recognize the value of co-production of knowledge [32]. We have engaged with knowledge users from the Canadian Diabetes Association, Ontario’s Ministry of Health and Long-Term Care, and Alberta Health Services (including relevant Strategic Clinical Networks). These knowledge users will help frame the specific research questions and participate in the interpretation of our results. We will convene three one-day, face-to-face meetings during the project. Knowledge user meeting 1, to be held in month 2, will aim to clarify the informational needs of decision-makers when making decisions regarding diabetes QI programs. We will present the results of the current review and explore its limitations from the perspective of decision-makers. Knowledge user meeting 2, to be held in month 15, will review the results from phases 1 and 2 and discuss progress with phase 3. We will use this meeting to identify potential additional information that we will collect during the survey of trial authors in phase 3. Specifically, we recognize that decision-makers require detailed information on potential effect modifiers (e.g., intervention components and contextual factors), and we will aim to extend the data set with information that can be used to address this need. Knowledge user meeting 3, to be held in month 32, will consider the results from phases 3 and 4 and assess whether the novel analytical approaches combined with the enriched and updated dataset enhance the utility of the systematic review. Our knowledge users will also be involved in the detailed planning for additional end-of-study KT activities (phase 5). In addition to the face-to-face meetings, we will update our knowledge users regularly by email and will convene additional meetings via teleconference, as judged necessary by the knowledge users or research team.

### Phase 1: additional analyses of the existing systematic review dataset using traditional meta-analytical approaches

As outlined above, the original systematic review [19,34] attempted to explore between-study heterogeneity by meta-regression across the 12 component QI intervention categories and subgroup analyses stratified by measures of baseline diabetes control (e.g., study-reported mean baseline HbA1c values). Prior to implementing novel meta-analytical approaches, we will conduct further exploratory subgroup and random effects meta-regression analyses to investigate whether intervention- or context-related factors can explain variation in the results. In exploring the current data set to its fullest using traditional meta-analytic approaches, we will be better equipped to fully assess the incremental utility of novel meta-analytic approaches. Meta-analyses will be conducted in Comprehensive Meta-analysis (version 2.2.050) and meta-regression analyses with SAS (version 9.2; SAS Institute, Cary, NC, USA) and OpenMeta-analyst (version 3, Center for Evidence-based Medicine, Brown University, Providence, RI, USA) [35,36].

### Phase 2: application of novel meta-analytical techniques to existing data

Using multivariate meta-regression analyses and all-subsets combinatorial meta-analyses, we will conduct both deductive and inductive analyses to assess the robustness of intervention categorization used in the systematic review and to explore potential sources of heterogeneity, respectively.

#### *Multivariate meta-regression analyses*

We will perform multivariate meta-regression analyses to estimate additive intervention effects, assess non-additive intervention effects, and evaluate effect modifiers [25-27]. The goals of the modeling will be first, to explore interactions between component interventions and second, to explore interactions between component interventions and potential effect modifiers. We plan to use a two-level structure to model the statistical distribution of data: trial arms (QI programs or usual care) are nested within RCTs. At the first level, we will model the outcome of interest (e.g., mean HbA1c value) within each arm of each RCT. The effect in each study arm will be regressed against the *K* = 13 management approaches of interest (12 QI component interventions plus usual care). At the second level, we will model between-study variability (heterogeneity).

To estimate the additive effects of component interventions on continuous outcomes (e.g., HbA1c values), we will use the following regression model where the outcome of interest, *y*_*ij*_ in arm *j* for study *i* is related to the presence or absence of the specific QI strategies (encoded by the indicator variables *x*_1*ij*_,…,*x*_*Kij*_):

$$y_{\text{ij}}=\overset{13}{\underset{k=1}{\sum }}\left(\beta_{k}+\zeta_{\text{ki}}\right)x_{\text{kij}}+\epsilon_{\text{ij}}$$

where *β*_1_,…, *β*_*K*_ are the *additive effects* of the *K* QI components across studies, (*ζ*_1*i*_,…,*ζ*_*Ki*_) ~ *MVN*(**0**, **T**) are the corresponding random effects, $\epsilon_{\text{ij}}\left|\zeta_{1i},\ldots ,\zeta_{\text{Ki}}\sim N\left(0,\sigma_{\text{ij}}^{2}\right)\right)$ are the residuals, and $\sigma_{\text{ij}}^{2}$ is the within-arm variance (conditional variance). The *K* × *K* covariance matrix of the random effects, **T**, expresses between-study heterogeneity for the *K* interventions of interest and must be estimated from the data; it is analogous to between-study heterogeneity (*τ*^2^) in univariate random effects meta-analysis. One can leave the covariance matrix **T** unstructured or impose structure to reduce the number of parameters that need to be estimated. The model can be extended to accommodate other types of outcomes (e.g., binary or count). Similarly, we may opt to modify the modeling strategy if the data structure requires us to do so. A detailed description of the random effects meta-regression model that we plan to use is available upon request.

The multivariate meta-regression model expresses the observed mean outcome of interest in each RCT arm as the combination of the effects of QI components (or usual care, as applicable). It assumes additive intervention effects, can be extended to include multi-arm RCTs as well as RCTs that compare only active QI programs, and accounts for unexplained between-study heterogeneity. Provided that no QI interventions are used always together, the model can estimate the effects of each QI component intervention of interest.

It is possible that combinations of QI component interventions interact. To assess non-additive intervention effects, we will extend the model described above. Up to 66 pairwise interactions can be formed between the 12 QI component interventions. We will assess these interactions (individually and in clinically relevant combinations) by including appropriate cross-product terms in the model. To assess potential effect modifiers of QI component interventions, we will extend the model by including additional regression terms. The number of predictors of interest is large and data may be sparse for such analyses. To avoid over-fitting and related issues, we may opt to use various model reduction strategies (e.g., assess only plausible interactions); simplify the categorization of interventions (e.g., define the component groupings more broadly based on substantive knowledge or group uncommon interventions into a single ‘mixed/other’ category); and use prior distributions to induce shrinkage of coefficients towards zero or informative prior distributions derived from empirical data (e.g., other meta-analyses of QI interventions) and expert opinion. All analyses will be conducted in Stata version SE/13.1 (Stata Corp., College Station, TX, USA) or JAGS [37].

#### *Identifying the most effective QI component interventions*

We will use the meta-regression analyses to rank QI component interventions according to their effectiveness and to identify the five most effective interventions in terms of their expected isolated effect size. The ranking of QI component interventions may be sensitive to outlier studies in the evidence base. We will examine its stability by a resampling procedure (i.e., bootstrapping). Specifically, we will generate a large number of datasets of size equal to the original dataset (142 studies) that have been sampled randomly with replacement from the original dataset (a few hundred to a thousand replications represent a good compromise between the need for stable estimates and feasible computation times). Subsequently, we will fit the selected hierarchical meta-regression model in the bootstrapped samples, and we will record the top five QI component interventions in each bootstrapped sample. We will then count how often each of the five most effective QI component interventions of the main analyses was included among the top five in the bootstrapped samples. The QI component interventions identified as more ‘robust’ in the bootstrapping and all-subsets combinatorial meta-analysis will be further examined in phase 4.

#### *All-subsets combinatorial meta-analyses*

For our previous review, we used an established, pragmatic taxonomy to identify the QI strategies used within complex interventions (Table 1). The categorization appears to have face validity given its acceptance within the field since it was first described in the Shojania review in 2006 [38]. Many of the individual QI strategies in the taxonomy have also been the subject of separate systematic reviews (e.g., audit and feedback, clinician reminders) [39].

**Table 1:** Taxonomy of knowledge translation/quality improvement intervention strategies

**Strategy**	**Operational definition**
Audit and feedback	Summary of clinical performance of health care delivered by an individual clinician or clinic over a specified period, transmitted back to the clinician (e.g., the percentage of a clinician’s patients who achieved a target HbA1c concentration or who underwent dilated-eye examinations with a specified frequency). This strategy is strictly based on clinical data and excludes clinical skills. It can include the number of patients with missing tests and dropouts.
Case management	Any system for coordinating diagnosis, treatment, or routine management of patients (e.g., arrangement for referrals, follow-up of test results) by a person or multidisciplinary team in collaboration with, or supplementary to, the primary care clinician. For a randomized controlled trial to qualify, the case management has to have happened more than once. If the study calls the intervention ‘case management,’ we classify it as such.
Clinician education	Interventions designed to promote increased understanding of principles guiding clinical care or awareness of specific recommendations for a target disorder or population of patients. Subcategories of clinician education include conferences or workshops, distribution of educational materials (e.g., written, video, or other), and educational outreach visits (i.e., academic detailing). We exclude teaching how to educate patients, counseling skills, motivational interviewing, self-directed learning, and skills related to the intervention (e.g., teaching how to use the website for the randomized controlled trial). We include all health care providers. If the education was part of the individual’s role (e.g., teaching a case manager about diabetes), we do not categorize it as clinician education.
Clinician reminders	Paper-based or electronic systems intended to prompt a health professional to recall patient-specific information (e.g., most recent HbA1c value) or to do a specific task (e.g., foot examination). If the strategy was accompanied by a recommendation, we subclassify it as decision support (e.g., giving targets to health care providers). An example is a yellow piece of paper clipped to the medical record with the patient’s information on it. This approach has to have been systematic and part of the implementation of the intervention—we exclude *ad hoc* clinician reminders.
Continuous quality improvement	Interventions explicitly identified as involving the techniques of continuous QI, total quality management, or plan-do-study-act, or any iterative process for assessing quality problems, developing solutions to those problems, testing their effects, and then reassessing the need for further action.
Electronic patient registry	General electronic medical record system or electronic tracking system for patients with diabetes. We do not include websites unless patients were tracked over time. To qualify, the system has to have been part of the clinical trial as an intervention (i.e., not pre-existing infrastructure unless used more actively).
Facilitated relay of clinical information	Clinical information collected from patients and transmitted to clinicians by means other than the existing medical record. We exclude conventional means of correspondence between clinicians. For example, if the results of routine visits with a pharmacist were sent in a letter to the primary care physician, the use of routine visits with a pharmacist counts as a ‘team change,’ but the intervention does not count as ‘facilitated relay.’ However, if the pharmacist issued structured diaries for patients to record self-monitored glucose values, which were then taken to office visits to review with the primary physician, we count the intervention as facilitated relay. Other examples include electronic or web-based methods through which patients provide self-care data and which clinicians reviewed, as well as point-of-care testing supplying clinicians with immediate HbA1c values. We include passports, referral systems, and dietary information (vs. purely clinical information). In general, the patient should be facilitating the relay. To be included, the information must have gotten to someone with prescribing or ordering ability. For example, if the nurse’s role was expanded to make drug changes, the patient had a portable personal record or ‘diabetes passport,’ and the nurse could directly make a change, we classify the intervention as case management and facilitated relay of clinical information (depending on the study and situation). If the nurse alerted the primary care provider that the patient had run out of drugs, we do not deem this facilitated relay of information because that is a normal part of a nurse’s role.
Financial incentives	Interventions with positive or negative financial incentives directed at providers (e.g., linked to adherence to some process of care or achievement of some target outcome). This strategy also includes positive or negative financial incentives directed at patients or system-wide changes in reimbursement (e.g., capitation, prospective payment, or a shift from fee-for-service to salary pay structure).
Patient education	Interventions designed to promote greater understanding of a target disorder or to teach specific prevention or treatment strategies, or specific in-person education (e.g., individual or group sessions with diabetes nurse educator, distribution of printed or electronic educational materials). Interventions with education of patients are included only if they also include at least one other strategy related to clinician or organizational change. We do not include occasions of optional education.
Patient reminders	Any effort (e.g., postcards or telephone calls) to remind patients about upcoming appointments or important aspects of self care (e.g., reminders to monitor glucose). Interventions with reminders are included only if they also included at least one other strategy related to clinician or organizational change. If the intervention included case management, patient reminders need to be explicit and to represent an extra task as compared to normal case management.
Promotion of self-management	Provision of equipment (e.g., home glucose meters) or access to resources (e.g., system for electronically transmitting home glucose measurements and receiving insulin dose changes based on those data) to promote self-management. Interventions promoting self-management are included only if they also include at least one other strategy related to clinician or organizational change. We also include established goals or a print off of a self-management plan (i.e., did not necessarily require equipment or resources). If the study called the intervention promotion of self-management, personalized goal-setting, or action-planning, it is included here. In general, we perceive this as a more active strategy than education of patients.
Team changes	Changes to the structure or organization of the primary health care team are defined as present if they meet the following criteria:
	1. Adding a team member or shared care—e.g., routine visits with people other than the primary physician (including physician or nurse specialists in diabetic care, pharmacists, nutritionists, podiatrists)
	2. Use of multidisciplinary teams—i.e., active participation of professionals from more than one discipline (e.g., medicine, nursing, pharmacy, nutrition) in the primary, routine management of patients
	3. Expansion or revision of professional roles (e.g., nurse or pharmacist had a more active role in monitoring of the patient or adjusting drug regimens). To ensure that every study we classify as case management does not also qualify as a team change, we classify a study of case management also as a study of team changes only if at least two of the above conditions are met. Team changes involve more communication. If the study called the intervention ‘joint visits’ or ‘shared care,’ we classify it as a team change. To qualify, the intervention has to have been done by a health care professional and has to have happened more than once.

We will use all-subsets combinatorial meta-analyses to deductively explore whether the taxonomy we used appears to identify clusters of homogeneous studies (e.g., clusters of studies sharing QI components, enrolling similar patient populations, or conducted in a the same care context). We will use the approach suggested by Olkin et al. to visualize the effect of study-level characteristics associated with differential treatment response [28]. This involves identifying the number of studies with a characteristic of interest in all of the subsets and exploring their distribution over all-subsets visually. All-subsets meta-analyses will be completed in Stata version SE/13.1 (Stata Corp., College Station, TX, USA); the all-subsets combinatorial method has been implemented as a C language plug-in for this software.

Of note, all-subsets combinatorial meta-analyses can become computationally challenging in large systematic reviews because of the large number of possible subsets. For the 142 studies included in the existing systematic review, the total number of subsets is approximately 5.6 × 10^42^. To overcome the associated computational limitations, we will implement a stratified sampling approach in the existing all-subsets algorithm to generate a sample of summary effect sizes representative of the overall distribution without the need to enumerate and analyze all possible combinations.

Our deductive all-subsets analyses will provide information about the robustness of the pragmatic QI taxonomy used in the existing review, potential interactions between pairs of QI component interventions, and potential effect modifiers. This information is exploratory and will complement the more formal analyses of interactions using multivariate meta-regression.

### Phase 3: updating and enriching the systematic review dataset

#### *Updating the dataset*

We estimate that at least 10–15 new RCTs of diabetes QI programs are published every year. Specifically, our search identified 16 new RCTs published between June 2009 and July 2010, the date of the last search for the current review. This suggests that by 2014, 40–60 new trials will have been published. We will update the systematic review following the methods adopted in the original review [19] to ensure that we have a complete and up-to-date dataset prior to phase 4.

#### *Enriching the dataset by surveying authors*

The utility of syntheses is highly dependent upon the quality of the data available in published studies. During the conduct of the original review, we noted that the key aspects of the interventions (e.g., who delivered the intervention, whether case managers could autonomously adjust medications), context (e.g., availability of electronic health records, characteristics of the patient population), and study design (e.g., allocation sequence generation, allocation concealment) were poorly reported [21]. Poor reporting might lead to misclassification of interventions, contextual factors, or study characteristics and reduce our ability to explore how intervention delivery or context influences the observed effects. Therefore, we intend to enrich the systematic review dataset by surveying authors of included studies about missing data elements. Research team members, including scientists and knowledge users, will collaborate to develop a questionnaire for study authors. The goal will be to prioritize variables related to intervention-design and context that are thought to be associated with effect size or feasibility of replication.

Recently, the members of our team undertook a web survey of authors of 300 randomly chosen cluster-RCTs published between 2000 and 2008 to assess ethical issues relevant to their trials and achieved a 64% response rate [40]. We will use similar methods to undertake a tailored web-based survey to contact authors of the included trials. Briefly, the details for the corresponding author will be abstracted from trial reports. Google™ *Web* and *Scholar* searches will be conducted to confirm current contact information and retrieve the corresponding author’s email address. If a trialist’s email address is not retrievable through Internet searches, a co-author on a recent publication will be contacted to obtain the trialist’s current email address. Our previous experience suggests that personalizing the request and providing the original study report as an attachment improves participation [40].

The mail merge feature in Microsoft Word® will be used to personalize the questionnaire by including details from our systematic review database specific to each trial; this will also permit us to avoid asking questions to authors who adequately reported the items of interest. Once finalized, the questionnaire will then be converted into a web survey using resources available at the Ottawa Hospital Research Institute (OHRI). The survey will have a unique identification number for each trial embedded in the survey link. Users will be able to review and change their responses using the ‘previous’ and ‘save and continue’ buttons featured at the bottom of each page. Once users submit their questionnaire, their unique identification numbers will be de-activated. Close-ended questions will be used to facilitate analysis, but free-text boxes will be placed throughout the questionnaire to allow respondents to clarify a specific response. We will also ask participants to share any materials used in their intervention as well as unpublished data and protocols. Prior to implementation, the web survey will be tested extensively by the research team. In addition to internal testing, we will conduct a pilot test of the web survey as recommended by Dillman et al. [41].

### Phase 4: analysis of updated and enriched diabetes systematic review dataset

Using all available data from the update and author survey, we plan to do analyses similar to those undertaken in the previous review and compare them with the novel approaches. Specifically, as in the original review and phase 1 of this project, the effects of each QI component on outcomes of interest will be analyzed descriptively. Random effects models will be used to estimate the pooled risk ratio (RR, dichotomous data) or mean difference (MD, continuous data) across included RCTs for all reported outcomes. Statistical heterogeneity will be qualitatively and quantitatively explored using forest plots and appropriate statistical measures (e.g., *I*^2^ index). Subgroup analyses will be conducted based on *a priori* hypotheses regarding contextual, program, or intervention characteristics thought to influence effectiveness. These will be based on the findings of exploratory analyses in phase 2 and discussions with knowledge users. Multivariate meta-regression analyses will be conducted for studies reporting HbA1c outcomes (and potentially systolic blood pressure and low-density lipoprotein cholesterol [LDL-c] levels, depending on the number of available studies for each of these outcomes), using the methods to be developed during phase 2 of this project. We will also conduct all-subsets combinatorial meta-analyses on the updated dataset to identify effective QI strategies and to illustrate the impact of effect modifiers. Finally, we will qualitatively compare and contrast the results obtained in phase 1 (traditional meta-analyses, subgroup analyses, and meta-regressions on published data), phase 2 (novel meta-analytical approaches on published data), and phase 3 (traditional meta-analyses, subgroup analyses, meta-regressions, and novel meta-analytical approaches on the enriched and updated data) with respect to their utility. Conducting traditional meta-analyses and subgroup analyses as well as using novel meta-analytical techniques on both enriched/updated and non-enriched/updated datasets will enable us to determine the relative yield of each approach.

### Phase 5: deliberative dialog with stakeholders

To facilitate interactions between the research team and a broader range of Canadian stakeholders, we will convene a deliberative dialog to consider the implications of the findings of our systematic review for the Canadian health system [42,43]. Deliberative dialogs provide unique support for evidence-informed decision-making by fostering the interplay of the best available data and research evidence with the tacit knowledge and views of those who will be involved in or affected by the decisions informed by the review findings. In preparation, we will produce an issue brief that draws on the best available data and from the research syntheses conducted in phases 1 through 4 of the project. The brief will aim to (1) define the problem(s) faced in Canada in diabetes management, (2) identify and describe what is known about possible QI program options for diabetes management, and (3) identify key implementation considerations for these options. We will invite 18–22 Ministry of Health or provincial health leaders, professional and community leaders, patients/citizens, and researchers. Following the dialog, we will prepare a dialog summary that captures participants’ tacit knowledge, views, and experiences as a complement to the brief. The issue brief and dialog summary will be disseminated to Canadian health system decision-makers through an evidence service run by the McMaster Health Forum as well as to the Canadian Diabetes Association Dissemination and Implementation Committee and Executive Leadership Team.

## Discussion

Complex interventions present unique challenges and opportunities for systematic reviews. Challenges arise due to the inherent complexity of primary studies included in syntheses of multi-component interventions. They necessitate refinement of the methods used by systematic reviewers to identify effective components of interventions and important effect modifiers. Advancing the methods of systematic reviews will permit more complex questions to be addressed and a richer, more nuanced knowledge base to be established.

Our review has produced a large dataset of studies assessing complex interventions for an important clinical condition. We will exploit this dataset to assess the added value of two innovative meta-analytic approaches with respect to increasing the utility of the review for health system decision-makers. We will also explore the utility of enriching the dataset via an author survey. Therefore, this study will contribute to the existing literature on knowledge synthesis of complex interventions by providing a greater understanding of diabetes QI programs and by evaluating several methodological tools that could be applied in similar reviews of complex interventions. In addition, the identification of modifiers of the effects of QI interventions could inform trial reporting frameworks [12] and the design of future trials.

## Abbreviations

KT: knowledge translation; MD: mean difference; QI: quality improvement; RCT: randomized controlled trial; RR: risk ratio.

## Competing interests

DM is the Founding Editor-in-Chief of *Systematic Reviews* journal.

## Authors’ contributions

The project was conceived by JMG and NI and developed with critical input from AT, TT, ID, DM, SS, BM, MT, and JNL. CY, AE, PS, MH, and AP contributed as knowledge users to refine the research questions. Analytical methods were developed by TT, ID, JMG, and TR. KJD drafted the manuscript and all authors critically revised it and approved the final version.

## Authors’ information

NI is a family physician at Women’s College Hospital, an Innovation Fellow with the WCH Institute for Health System Solutions and Virtual Care. JMG is a Senior Scientist in the Clinical Epidemiology Program at the Ottawa Hospital Research Institute and a Full Professor in the Department of Medicine at the University of Ottawa. ACT is a Scientist at the Li Ka Shing Knowledge Institute at St. Michael’s Hospital in Toronto. TAT is an Associate Professor of Health Services, Policy & Practice and Director of the Center for Evidence-Based Medicine at Brown University. IJD is an Assistant Professor of Health Services, Policy & Practice and founding member of the Centre for Evidence-Based Medicine at Brown University. KJD is a Research Coordinator at the Ottawa Hospital Research Institute. DM is a Senior Scientist at the Ottawa Hospital Research Institute and a Research Chair at the University of Ottawa. SES a Scientist at the Keenan Research Centre of the Li Ka Shing Knowledge Institute at St. Michael’s Hospital in Toronto and Director of the Knowledge Translation program at the Li Ka Shing Knowledge Institute and University of Toronto. JNL is a Professor in the Department of Clinical Epidemiology & Biostatistics at McMaster University. He is the Director of the McMaster Health Forum and the Program in Policy Decision-Making, the Associate Director of the Centre for Health Economics and Policy Analysis, and a member of the Department of Political Science at McMaster. He is also an Adjunct Professor in the Department of Global Health and Population, Harvard School of Public Health. CHY is an Associate Scientist at the Li Ka Shing Knowledge Institute at St. Michael’s Hospital in Toronto, Staff Endocrinologist at St. Michael’s Hospital, and Chair of the Dissemination and Implementation Committee of the Canadian Diabetes Association 2013 Clinical Practice Guidelines. KS is a Scientist at Sunnybrook Research Institute and Director of the Centre for Quality Improvement and Patient Safety at the University of Toronto. BM is a Professor at the University of Calgary in the Departments of Medicine & Community Health Sciences. He is the Incoming President of the Canadian Society of Nephrology. MT is a Professor at the University of Calgary in the Division of Nephrology. He is the Chair of the Canadian Task Force for Preventative Health Care. TR is a Scientist in the Clinical Epidemiology Program at the Ottawa Hospital Research Institute and Assistant Professor at the University of Ottawa in the Department of Epidemiology and Community Medicine in the Faculty of Medicine. AE is a Professor at the University of Calgary in the Division of Endocrinology and Metabolism and the Senior Medical Director for the Strategic Clinical Network for Diabetes, Obesity and Metabolism with Alberta Health Services. PS is the Medical Director of Chronic Disease Management with Alberta Health Services. AP was the Director of Planning, Research and Analysis Branch at Ministry of Health and Long-Term Care Ontario. AP is currently Interim Director, Partnerships, Ontario SPOR SUPPORT Unit.
